# “It’s easier in pharmacy”: why some patients prefer to pay for flu jabs rather than use the National Health Service

**DOI:** 10.1186/1472-6963-14-35

**Published:** 2014-01-24

**Authors:** Claire Anderson, Tracey Thornley

**Affiliations:** 1 School of Pharmacy, University of Nottingham, NG72RD Nottingham, UK; 2 Boots UK Ltd and Honorary Lecturer School of Pharmacy, University of Nottingham, Nottingham, UK

**Keywords:** Influenza, Vaccination, Immunisation, Community pharmacy, Risk groups, Patient group directions, Private

## Abstract

**Background:**

There is a need to increase flu vaccination rates in England particularly among those under 65 years of age and at risk because of other conditions and treatments. Patients in at risk groups are eligible for free vaccination on the National Health Service (NHS) in England, but despite this, some choose to pay privately. This paper explores how prevalent this is and why people choose to do it. There is moderate to good evidence from several countries that community pharmacies can safely provide a range of vaccinations, largely seasonal influenza Immunisation. Pharmacy-based services can extend the reach of immunisation programmes. User, doctor and pharmacist satisfaction with these services is high.

**Method:**

Data were collected during the 2012–13 flu season as part of a community pharmacy private flu vaccination service to help identify whether patients were eligible to have their vaccination free of charge on the NHS. Additional data were collected from a sample of patients accessing the private service within 13 pharmacies to help identify the reasons patients paid when they were eligible for free vaccination.

**Results:**

Data were captured from 89,011 privately paying patients across 479 pharmacies in England, of whom 6% were eligible to get the vaccination free. 921 patients completed a survey in the 13 pharmacies selected. Of these, 199 (22%) were eligible to get their flu vaccination for free. 131 (66%) were female. Average age was 54 years. Of the 199 patients who were eligible for free treatment, 100 (50%) had been contacted by their GP surgery to go for their vaccination, but had chosen not to go. Reasons given include accessibility, convenience and preference for pharmacy environment.

**Conclusions:**

While people at risk can access flu vaccinations free via the NHS, some choose to pay privately because they perceive that community pharmacy access is easier. There are opportunities for pharmacy to support the NHS in delivering free flu vaccinations to patients at risk by targeting people unlikely to access the service at GP surgeries.

## Background

There is a recognised need to continue driving up flu vaccination rates in England particularly among those under 65 years of age and at risk because of other conditions and treatments such as: chronic respiratory disease, chronic heart disease, diabetes, being immunocompromised as well as pregnant women and frontline health and social care workers. By the end of the 2011/12 flu vaccination season, just over 50% of people under 65 years in clinical risk groups had been vaccinated against flu. Increasing uptake is important because of the increased risk that people in risk groups are at from the effects of flu. Achieving this level of uptake is challenging and requires innovative thinking and new approaches to deliver these changes in outcomes. The long term National Health Service (NHS) flu vaccination target in England is to achieve a 75% vaccine uptake among the under-65 s in a clinical risk group by 2013/14. The NHS were asked to achieve a target of 70% uptake in 2012/13 for that group. For those 65 years of age and over, the target was to achieve 75% uptake 2012/13 [1]. The NHS recognise that community pharmacies are increasingly providing both NHS and non-NHS immunisation services. Each year, many local pharmaceutical committees across the country present a case to their local National Health Service commissioners for using community pharmacy to provide seasonal ‘flu vaccinations. Over the last four years, an increasing number of Local Pharmaceutical Committees have been successful and some have gathered data on the delivery of the service [2]. Patients in at risk groups are eligible to get a free vaccination on the NHS in England, but despite this, some choose to pay privately. This paper explores how prevalent this is and the reasons why people choose to do it. Community pharmacy has the opportunity to support the NHS in delivering free flu vaccinations to patients at risk using patient group directions (PGDs). Patient Group Directions (PGDs) are written instructions for the supply or administration of medicines to groups of patients who may not be individually identified before presentation for treatment. PGDs have become an important way of providing and administering medicines to patients. They are an integral part of the new ways of working for pharmacy and other healthcare professionals in both the NHS and private sector. A PGD was used for the private service which was signed off by the Medical Director and Superintendent Pharmacist of the pharmacy chain. The PGD for the NHS service was agreed within each Primary Care Trust (PCT) and signed by the relevant PCT medical director and chief pharmacist. Many of the target groups use pharmacies to either collect a prescription or seek advice. Pharmacies offer convenience, particularly around working hours and at weekends when many GP surgeries are closed. They are located near to where people work or shop, with large patient catchment areas. Pharmacists can also alert patients who are in at-risk groups of the need for vaccination, even if they are not administering the vaccines themselves. This is particularly relevant for people who do not routinely visit GP surgeries.

Two major literature reviews indicate that there is moderate to good evidence from several countries that community pharmacies can safely provide a range of vaccinations, largely seasonal influenza immunisation [3,4].

This is particularly evident in the United States. Services can be provided safely through community pharmacies [5]. In the US pharmacists and student pharmacists are formally trained through recognized programs as vaccine experts, and the practice of pharmacist-administered immunisations, particularly for adult patients, has become routinely accepted as an important role of the pharmacist [6]. Pharmacy patient medication records are effective in identifying ‘at risk’ clients who can then be invited for immunisation [7] and pharmacy-based services can extend the reach of immunisation programmes [8]. User satisfaction with these services is high and support for non-physician immunisation was found to be greater for adult than for child immunisation [8,9]. A UK pharmacy-based immunisation service (for influenza in particular) seems to have been reasonably well accepted by patients, physicians and pharmacists [10]. Using pharmacies for flu immunisation in the US where access to doctors is very costly has been shown to be cost saving or relatively cost saving even for healthy adults [11]. A recent US study [12] that examined retrospective data from flu immunisations in 2009–10 in the Walgreens chain indicates that community pharmacies are convenient and accessible venues at which patients can obtain seasonal influenza vaccines. Pharmacies were well-positioned to deliver these services, particularly in areas that are otherwise medically underserved. Educational interventions by the community pharmacists have also been shown to encourage greater patient knowledge and uptake of vaccination [13,14].

The uptake of NHS-commissioned flu vaccinations in community pharmacies in England has increased significantly in recent years, due in part to published data on the success of locally driven services. The Sheffield Local Pharmaceutical Committee published an evaluation of the 2011–12 flu vaccination ‘mop-up’ programme for hard to reach ‘at-risk’ groups aged 18–65 [15]. 170 vaccinations were administered by 12 pharmacies located in a variety of settings including high streets, supermarkets, health centres, close to GP surgeries and in town and city centres. 61% of patients cited convenience as the reason for selecting community pharmacy as a location for receiving their seasonal flu vaccination. Also mentioned were ‘no appointment needed’, ‘easy access’ or ‘no waiting queue’. 36% of patients said they would not have had the vaccination if they had not had it offered and administered in the pharmacy. The pharmacy service did not impact on the uptake of flu vaccinations from GP surgeries: there was no decrease in GP vaccination rates in the area [16].

## Methods

This paper discusses retrospective data collected through a sample of Boots UK community pharmacies during 2012–13 to help support the case for national commissioning of flu vaccinations through pharmacy. Boots UK operates the largest chain of community pharmacies in the United Kingdom. There are approximately 2,500 pharmacies trading under the Boots brand in the UK and these are well distributed across the country. The chain encompasses those which serve small local communities, including some of the most deprived locations in the country, and health centres through to high streets and those that are part of the largest retail and destination shopping centres. Boots UK is a member of Alliance Boots, a leading international pharmacy-led health and beauty and pharmaceutical wholesaling group.

The majority of flu vaccinations administered through community pharmacy are currently private rather than NHS. During the 2012–13 flu season these vaccinations were available in 586 participating Boots UK pharmacies in England at a cost of £12.99 to the patient [14]. Boots UK were also commissioned to deliver the NHS service via a Patient Group Direction (PGD) within 258 of these 586 pharmacies.

Pharmacist training consisted of pre reading materials, face to face training on vaccination techniques, counselling skills, and an e learning module. Each pharmacist then undertook a sign off process for being competent to provide the service.

Each patient attending for a privately paid flu vaccination within a Boots UK pharmacy has to provide some basic information listed within the flu leaflet (such as gender and date of birth). Patients fill in the first part on their own and then the pharmacists fills in the second section with the patient. The information is recorded on the form and kept as a paper record copy in the pharmacy. Some of the data is then captured electronically as part of the PGD process. Pharmacists discussed NHS eligibility criteria with patients, and where they were eligible, advised them that they could access the vaccination for free on the NHS through their GP surgery.

Additional data were collected from a sample of patients accessing the private service within 13 purposively sampled pharmacies. The pharmacies were were selected from those already providing the private service, and ensuring a representative mix of city centre and edge of town pharmacies. Pharmacies delivering the NHS commissioned service were avoided. Pharmacies from the top 100 performing pharmacies (based on flu performance) were chosen to help identify reasons for choosing to pay privately through community pharmacy. As it was additional workload, pharmacies area managers had to agree to the pharmacy participating. Pharmacies offering the free NHS flu service were avoided.

Every patient who had a vaccination was approached with a questionnaire (see Additional file 1) between 24^th^ September and 7^th^ December 2012, but not all chose to complete. The questionnaire had already been used during the previous year. The anonymous data were collected as part of Boots normal processes as a service review and no ethics approval was necessary.

## Results

During 2012–13, private flu vaccinations were delivered in 586 Boots UK pharmacies across England. 258 Boots UK pharmacies (a subset of 586) were also commissioned to provide NHS flu vaccinations to eligible patients. This represents 44% of Boots UK pharmacies delivering the private and NHS flu service. PGD data were captured from 89,011 privately paying patients across 479 pharmacies, of which 6% were eligible to get the vaccination free on the NHS.

Within the sample of 258 pharmacies, NHS flu vaccinations accounted for 11% of all vaccines administered up to 16^th^ March 2013. NHS flu vaccinations represented 5% of all Boots UK flu vaccinations in total.

479 of the 586 pharmacies (82%) collected information on the reasons for NHS eligibility for those patients opting to pay for their flu vaccination privately. This was captured through the patient Flu Jab Service Leaflet and the online PGD form. Data were not collected on NHS eligibility in all pharmacies. Pharmacists were not 100% compliant in recording the eligibility data on the electronic PGD form as this information was not compulsory. However, of those that did collect the data, the sample represents 86% of private flu vaccinations conducted in those pharmacies. Private-only pharmacies were more compliant in providing the data electronically than those commissioned to also provide the NHS service.

Eligibility data on 89,011 patients were captured and recorded online up to 16^th^ March 2013. 5,323 of these (6%) were recorded as being eligible for the free NHS flu vaccination. Of these 2,823 patients (53%) were aged 65 or over. 1,448 patients (27%) were under 65 but considered ‘at risk’. No reason was given for 1,052 patients (20%). Within the Boots UK pharmacies that provided the NHS-commissioned service, 1,811 patients (5%) were eligible for free vaccinations but still chose to pay privately (data were captured in 175 out of the 258 pharmacies providing the NHS service).

**Figure 1:**
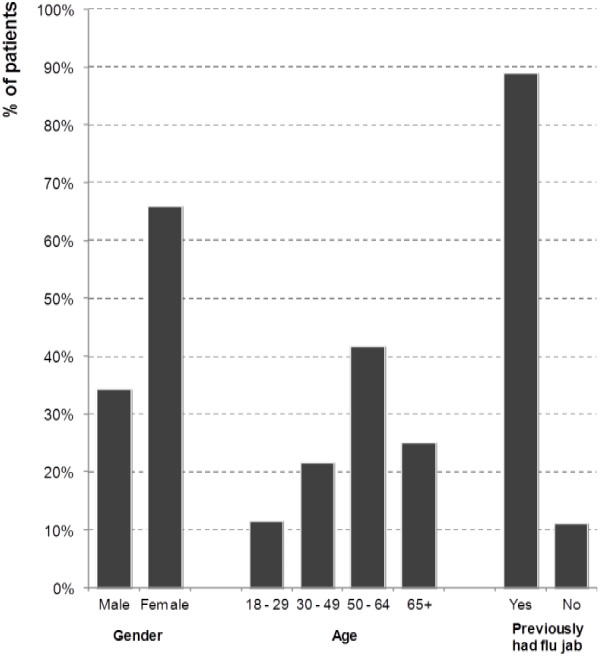
Demographic details of 199 NHS eligible patients accessing the private service.

Additional survey information was collected from 921 privately-paying patients in the sample of 13 pharmacies. The 921 patients interviewed represented 30% of all private flu vaccinations administered in the 13 pharmacies selected. Of these, 199 (22%) were eligible to get their flu vaccination for free on the NHS. 131 (66%) were female. The average age of an eligible patient was 54 years. 133 (67%) were aged at least 50, with 50 (25%) being 65 or over. 9 in 10 eligible patients (177, 89%) had previously had a flu vaccination, 53% at a pharmacy, 49% at their GP surgery and 3% at work (Figure 1).

Of the 199 patients who were eligible for free NHS treatment, 100 (50%) had been contacted by their GP about going into their local surgery for their vaccination, but had chosen not to. The reasons given are largely because of accessibility and convenience and are summarised in Table 1. The 199 patients eligible for the free NHS vaccination met the eligibility criteria in Table 2. The key reasons given for visiting a pharmacy by the eligible population (199) are again largely around accessibility and convenience and are summarised in Table 3. The key reasons given for visiting a pharmacy by all patients (921) are summarised in Table 4.

**Table 1:** Reasons given for not visiting a GP surgery to get a flu immunisation based on the patients who were contact by their GP (sub set survey sample, n = 100)

Reason given for not visiting a GP**	**Number of patients**	**Proportion of patients (n = 100)**
Not convenient to go there	44	44%
Difficult to get an appointment	34	34%
Vaccine unavailable	21	21%
Prefer to go to a pharmacy	15	15%
Other*	18	18%
Total responses	132	

*Other reasons given included not eligible at the surgery (10), unable to access GP (2), patients not wanting to wait (2), patient referred to a pharmacy (1), flu jab batch failures (1), limited flu clinics (1), surgeries considered too crowded (1).

**Note that some patients gave more than one reason, and therefore the total responses (132) exceed the number of patients who responded to this question (100).

**Table 2:** Reasons for eligibility for a flu immunisation (survey sample n = 199)

Eligibility criteria*	**Number of patients**	**Proportion of total (n = 199)**
Have a long-term condition	53	27%
*Asthma*	*39*	*20%*
*Diabetes*	*6*	*3%*
*COPD*	*5*	*3%*
*Cancer*	*2*	*1%*
*Splenectomy*	*1*	*1%*
Aged 65 years or over	48**	24%
Care for somebody with a long-term condition	34	17%
Frontline health worker	28	14%
Pregnant	7	4%
Not known	35	18%
Total responses	258	

• Note that some patients satisfied more than one criteria and therefore the total responses (258) exceed the number of patients who responded to this question (199).

**In relation to the number of people aged 65+ for the n = 50 in the text and n = 48 in Table 2 as people had free choice to chose their reason for eligibility and 2 may have chosen a reason other than age.

**Table 3:** Reasons for visiting a pharmacy for a flu immunisation (those patients eligible for free NHS immunisation that completed the survey, n = 199)

Reason for visiting a pharmacy**	**Number of patients**	**Proportion of total (n = 199)**
Convenient location	101	51%
Convenient opening hours	85	43%
Prefer the pharmacy environment and/or staff	50	25%
Inconvenient getting to a surgery	37	19%
Vaccine available	23	12%
Saw the service advertised in-store	20	10%
Spur of the moment decision	8	4%
Employer-purchased voucher	8	4%
Staff member	3	2%
GP referral	3	2%
Trust pharmacist	3	2%
Other*	22	11%
Total responses	363	

*Other reasons given included GP appointment availability, surgery waiting times, pharmacist recommendation and the pharmacy being less expensive than elsewhere.

**Note that some patients gave more than one reason and therefore the total responses (363) exceed the number of patients who responded to this question (199).

**Table 4:** Reasons for visiting a pharmacy (all patients completing the survey, n = 921)

Reason for visiting a pharmacy**	**Number of patients**	**Proportion of total (n = 921)**
Convenient location	542	59%
Convenient opening hours	399	43%
Prefer the pharmacy environment and/or staff	207	22%
Saw the service advertised in-store	127	14%
Inconvenient getting to a surgery	115	12%
Employer-purchased voucher	51	6%
Spur of the moment decision	29	3%
Vaccine available	24	3%
Staff member	16	2%
Previous experience	13	1%
Trust pharmacist	12	1%
Not eligible at their GP	11	1%
Other*	94	10%
Total responses	1640	

*Other reasons given included patient referred to a pharmacy, the pharmacy being less expensive than elsewhere, more appointments available, surgery waiting times, pharmacist recommendation and word of mouth.

**Note some patients gave more than one reason and therefore the total responses (1640) exceed the number of patients who responded to this question (921).

## Discussion

The data indicates that there are opportunities for pharmacy to support the NHS in delivering free flu vaccinations to patients at risk by targeting those patients unlikely to access the service at GP surgeries. By commissioning these services through community pharmacy, data on these patients would help contribute towards NHS/World Health Organisation (WHO) targets.

There are a number of patients that are eligible for the NHS flu vaccination, but choose to pay privately to access the service through community pharmacies. Reasons for not accessing the service from the GP include difficulty in getting an appointment and inconvenience. Like previous studies [6-8,10] reasons for accessing services through pharmacies include convenient locations, opening hours, and preference for the pharmacy environment.

Even though these patients could access the flu service for free at GP surgeries, they actively chose to go into pharmacy and pay privately. This was because of the inconvenience of going to GP surgeries (opening hours, appointments) and the convenience of pharmacies (hours, locations, accessibility). It is interesting that only 50% of the eligible people in this study recalled being contacted by their GP regarding flu vaccination. Some of this could be due to recall bias but it is concerning if 50% of people are genuinely not being contacted.

This represents a major missed opportunity for the NHS as they are not currently meeting their targets for vaccinations. There is a huge opportunity for pharmacy to augment the service that GPs currently provide, and help fill this gap. Pharmacists often see patients monthly (sometimes more frequently) when coming in to access their prescriptions. Pharmacists and their staff have an opportunity to remind patients in at risk groups to get vaccinated. Patients (particularly those under 65) may not see their GP on a regular basis, so may not get any reminders for vaccinations, or easily forget.

In other reported studies 23 Isle of Wight pharmacies vaccinated 4,192 patients in 2011–12. 65% were aged 65 or over, indicating that a third of patients were in the under 65 ‘at risk’ groups, particularly those with heart disease, diabetes or respiratory disease [17]. 71% of patients visited pharmacy because of its convenient accessibility, 16% indicated a general preference for pharmacy and 13% cited difficulties in obtaining vaccination services from their GP. 19% of patients would not have received a vaccination other than in a pharmacy. 99% of the Sheffield patients said they considered the service provision by the pharmacy good or excellent [15]. The Isle of Wight pharmacy service was rated excellent by 97% of patients.

### Limitations

The data presented are limited to one pharmacy multiple. The data are limited to information collected on a PGD record form and entered into a computer system. This limits other information commonly associated with immunisations such as patient health status.

The difference in the reported number of eligible patients between the PGD data and the survey data is likely due to the bias in collecting the survey data. Information was captured on 82% of patients having the flu vaccination within 479 pharmacies. Only 30% of patients completed the survey data within 13 pharmacies this further limits the findings. The pharmacists may have selected patients who were more likely to be NHS eligible for completing the survey, leading to bias in reporting of the higher percentage eligibility data.

## Conclusions

The data indicates that there are opportunities for pharmacy to support the NHS by targeting those patients unlikely to access the service at GP surgeries by offering both NHS and private immunisations.

## Competing interests

TT is an employee of Boots UK Ltd. CA has not received reimbursement, fees, funding or a salary from Boots UK Ltd in the past five years.

## Authors’ contributions

TT collected and processed the data. CA and TT wrote the paper. The study was funded by Boots UK. CA received no payment for writing the paper. We acknowledge the pharmacists who collected the data for us. All authors read and approved the final manuscript.

## Pre-publication history

The pre-publication history for this paper can be accessed here:

http://www.biomedcentral.com/1472-6963/14/35/prepub

## Supplementary Material

Additional file 1 Flu vaccination data collection (England 2012). Click here for file
