# Supplementary material for: “It’s easier in pharmacy”: why some patients prefer to pay for flu jabs rather than use the National Health Service
Source: BMC Health Serv Res. 2014 Jan 24;14:35. doi: 10.1186/1472-6963-14-35 (PMC3902185; doi:10.1186/1472-6963-14-35)
Supplement: Additional file 1 — Flu vaccination data collection (England 2012). [file 1472-6963-14-35-S1.docx]

**Flu vaccination data collection (England 2012)**

**Pharmacist to record:**

a. Store number: _________________

b. Customer gender: Male Female

c. Customer age: ________________

1. **Has your GP contacted you about going into the surgery to get a flu jab?**Yes  *(ASK Q2; THEN Q3)*
   No  *(SKIP Q2)*Don’t know  *(SKIP Q2)*

*(ASK IF Q1 WAS YES)*

1. **Why have you chosen not to get a flu jab from the GP?**Difficult to get an appointment
   Not convenient to visit the surgery
   Prefer to come into the pharmacy

Not eligible for the flu jab at the surgery
Other (please specify) __________________________________________

1. **Why have you decided to come to the pharmacy to get your flu jab?**Convenient opening hours
   Convenient location
   Inconvenient getting to a surgery

Saw the service advertised in-store

Prefer the pharmacy environment and/ or staff
Spur of the moment decision
Other (please specify) __________________________________________

1. **Do any of the following apply to you?**I have a long-term medical condition  *(ASK Q5)*

I am pregnant

I am aged 65 years or over

I am the carer of someone with a medical condition

I am a frontline healthcare worker
 None of these

*(ASK IF Q4 WAS YES)*

1. **What long-term medical condition do you have?**___________________________________________________________
2. **Have you previously had a flu jab?**

Yes  *(ASK Q7)*

No

1. *(ASK IF Q6 WAS YES)*

**Where did you have this done?**

In a GP surgery

At a pharmacy

At work
Other (please specify) ______________________________________
